# Teager Energy Entropy Ratio of Wavelet Packet Transform and Its Application in Bearing Fault Diagnosis

**DOI:** 10.3390/e20050388

**Published:** 2018-05-21

**Authors:** Shuting Wan, Xiong Zhang

**Affiliations:** Department of Mechanical Engineering, North China Electric Power University, Baoding 071003, China

**Keywords:** bearing diagnosis, kurtogram, WPT, TEER

## Abstract

Kurtogram can adaptively select the resonant frequency band, and then the characteristic fault frequency can be obtained by analyzing the selected band. However, the kurtogram is easily affected by random impulses and noise. In recent years, improvements to kurtogram have been concentrated on two aspects: (a) the decomposition method of the frequency band; and (b) the selection index of the optimal frequency band. In this article, a new method called Teager Energy Entropy Ratio Gram (TEERgram) is proposed. The TEER algorithm takes the wavelet packet transform (WPT) as the signal frequency band decomposition method, which can adaptively segment the frequency band and control the noise. At the same time, Teager Energy Entropy Ratio (TEER) is proposed as a computing index for wavelet packet subbands. WPT has better decomposition properties than traditional finite impulse response (FIR) filtering and Fourier decomposition in the kurtogram algorithm. At the same time, TEER has better performance than the envelope spectrum or even the square envelope spectrum. Therefore, the TEERgram method can accurately identify the resonant frequency band under strong background noise. The effectiveness of the proposed method is verified by simulation and experimental analysis.

## 1. Introduction

The rolling bearing is one of the most common components in rotating machinery, and it is also one of the components with the highest failure rate. The running condition of bearings is directly related to the safe and stable operation of the whole rotating machinery system. Extracting fault feature information from bearing vibration signals under strong background noise is a hot issue in the field of mechanical fault diagnosis. The process of bearing fault diagnosis is divided into two parts: vibration parameter (displacement, velocity and acceleration) acquisition and vibration data processing. Adamczak et al. analyzed the existing bearing fault testing system. They strived for more accurate bearing fault measurement equipment and fault measurement parameters by comparing and analyzing existing measuring devices [1]. Meanwhile, a large number of vibration signal processing methods have been put forward, among which the kurtogram method is able to adaptively recognize the resonance frequency band of the vibration signal. By filtering the resonance frequency band and integrating the spectrum analysis of filtered signals, the fault characteristics of vibration signals can be effectively extracted.

Kurtosis is the 4th-order cumulant of random variables. This index cannot reflect the change of specific signals, and is not suitable for condition monitoring under strong noise environments. In order to overcome the shortage of kurtosis in engineering applications, Antoni made a formal definition of kurtosis [2]. Subsequently, Antoni further proposed the kurtogram. The core idea of the algorithm is that the spectral kurtosis index of the unstable signal is maximized by reasonable selection of frequency resolution [3]. In order to reduce the operation time of the kurtogram and enable it to be widely used in engineering practice, Antoni further proposed a fast spectral kurtosis (FSK) calculation method based on a fast algorithm for computing the kurtogram over a grid. FSK can simplify the process of selecting the optimal band (f,Δf) [4]. In the FSK algorithm, the horizontal axis represents the frequency, the vertical axis represents the number of decomposition layers, and the depth of the color indicates the spectral kurtosis value of each sub band.

Since then, many algorithms based on the kurtogram have been proposed. Lei et al. proposed an improved kurtogram method [5], which uses wavelet packet transform (WPT) instead of the short time Fourier transform (STFT) and FIR filters used in the traditional kurtogram. A filter based on WPT can accurately divide the frequency bands and control the noise effectively [5]. Then, on the basis of Lei et al., Wang et al. made further improvements. The kurtosis calculation method in the traditional kurtogram was changed to calculate the power spectrum of the envelope of different nodes of WPT. The power spectrum of the envelope signal reflects the sparsity of the signal and helps to capture the resonance band in the signal [6]. Based on the same idea, Tse and Wang proposed a new method called sparsogram. The power spectrum of the envelope was applied to improve the kurtosis index in the kurtogram, so that it reflected the sparsity of signals [6,7]. Gu proposed a method for analyzing kurtograms in the frequency domain [8]. The correlated kurtosis of the envelope was used to replace the traditional calculating method in the kurtogram, which makes the calculation index sensitive to periodic impulses and has better robustness. To obtain the correct relationship between the node and frequency band in WPT, a vital process called frequency ordering is conducted to solve the frequency folding problem due to down-sampling. Tian et al. proposed the modulation signal bispectrum (MSB)-based robust detector to reduce the noise and accurately identify the resonance frequency band [9]. The high-magnitude features that result from the use of the MSB also enhance the modulation effects of a bearing fault. In gearboxes, the kurtogram method would fail when gears and bearings partially malfunctioned at the same time. In view of this, Wang proposed an improved method by combining kurtogram and menshing resonance (MRgram) [10]. Random impulses often occur in the frequency bands of vibration signals of bearings, making the selection of kurtogram resonance frequency bands inaccurate. Xu et al. proposed a periodicity-based kurtogram to deal with random impulse resistance [11]. The periodic component to aperiodic component ratio (PAR) was utilized in this method to differentiate the types of impulses. Li et al. improved the kurtogram based on an impulse step dictionary and a reweighted minimizing nonconvex penalty Lq regular for rolling bearing fault diagnosis [12].

Entropy was originally a thermodynamic concept describing the regularity of information. Because entropy has some statistical properties superior to kurtosis in some respects, the application scope of entropy was gradually extended to the field of mechanical fault diagnosis. Antoni introduced Shannon entropy into the kurtogram algorithm and proposed infogram [13]. Infogram contains three parts, the envelope (SE) infogram, squared envelope spectrum (SES) infogram, and SE_1/2_/SES_1/2_ infogram. The calculation index of the SE infogram is the negative entropy of the square envelope, and the calculation index of the SES infogram is the negative entropy of the square envelope spectrum. On the basis of infogram, the multiscale clustering grey infogram (MCGI) was proposed by Li et al. [14]. Hemmati et al. proposed an index that combines kurtosis and Shannon entropy, and the index was used to obtain the optimal band pass filter utilizing wavelet packet transform (WPT) and envelope detection [15]. The MCGI combined the negentropies of the time and frequency domains in a grey fashion using multiscale clustering. Chen et al. proposed a fault classification method combining time-spectral kurtosis, entropy and support vector machines (SVM). Combining the advantages of time-spectral kurtosis (T-SK) and entropy, this method makes fault extraction more obvious [16].

To sum up, the improvement of kurtogram has mainly focused on two aspects. On the one hand, the decomposition methods (STFT, FIR) of the traditional kurtogram algorithm have been improved, so that the frequency band segmentation will have better results. On the other hand, the calculation index of each sub-band has been improved. The aim is to make the calculation index sensitive to the periodic impact caused by the fault, and to have strong robustness to the interference caused by noise or accidental impact. These improvements have made a significant contribution in some respects. In order to further improve the accuracy and efficiency of the algorithm in determining the center frequency and bandwidth, a new adaptive method called TEERgram is proposed in this article.

## 2. The Index of Teager Energy Entropy

In 2016, Antoni proposed the infogram method. Infogram theories include the squared envelope (SE) infogram, the squared envelope spectrum (SES) infogram, and the SE_1/2_/SES_1/2_ infogram. The SE and SES were defined as Equations (1) and (2).

(1) $$\Delta I_{e}=\frac{\mathrm{SE}{\left(n;f,\Delta f\right)}^{2}}{\langle \mathrm{SE}{\left(n;f,\Delta f\right)}^{2}\rangle }\mathrm{In}\left(\frac{\mathrm{SE}{\left(n;f,\Delta f\right)}^{2}}{\langle \mathrm{SE}{\left(n;f,\Delta f\right)}^{2}\rangle }\right)$$

(2) $$\Delta I_{E}=\frac{\mathrm{SES}{\left(n;f,\Delta f\right)}^{2}}{\langle \mathrm{SES}{\left(n;f,\Delta f\right)}^{2}\rangle }\mathrm{In}\left(\frac{\mathrm{SES}{\left(n;f,\Delta f\right)}^{2}}{\langle \mathrm{SES}{\left(n;f,\Delta f\right)}^{2}\rangle }\right)$$

where n∈Z, f is the frequency, and Δf is the frequency resolution. $\Delta I_{e}$ and $\Delta I_{E}$ are entropy values in the time domain and entropy in the frequency domain respectively. That is, they were used to represent the impact characteristics and cyclostationarity characteristics of fault signals, respectively. Antoni further proposed the SE_1/2_/SES_1/2_ infogram to characterize the impact characteristics and cyclostationarity. The SE_1/2_/SES_1/2_ infogram was defined by Equation (3):

(3) $$\Delta I_{1/2}=\Delta I_{e}/2+\Delta I_{E}/2$$

### 2.1. Teager Energy Operator

Teager energy operator (TEO) can enhance transient impact components, which is suitable for detecting impact characteristics in signal, and has good effect in fault impact feature extraction. For signal x(t), the Teager energy operator ψ can be defined as Equation (4):

(4) $$\psi \left(x\left(t\right)\right)={\left(x\prime \left(t\right)\right)}^{2}-x\left(t\right)x^{\prime \prime }\left(t\right)$$

where x′(t) and x″(t) are the first- and second-order derivatives of signal *x* relative to time *t*. The general expression of the amplitude modulation frequency modulation signal is shown in Equation (5):

(5) s(t)=a(t)cos(ψ(t))

where *a(t)* is the instantaneous amplitude of s(t) and ψ(t) is the instantaneous phase of s(t). The instantaneous frequency of the signal s(t) is ω = dψ/dt. The effect of the energy operator on s(t) is shown in Equation (6):

(6) $$\psi \left(s\left(t\right)\right)=\left(a\left(t\right)\psi^{\prime }\left(t\right)\right)/2+a^{2}\left(t\right)\psi \left(t\right)\times \sin\left(2\psi \left(t\right)\right)/2+\cos^{2}\left(\psi \left(t\right)\right)\psi \left(a\left(t\right)\right)$$

Because the change of the carrier signal is much faster than the modulated signal, the instantaneous amplitude and the instantaneous frequency of the modulated signal are relatively slow relative to the high-frequency carrier, and can be approximately equal to the constant. Therefore, if $\psi^{\prime \prime }\left(t\right)\approx 0$, ψ(a(t))≈0, then the Formula (6) can be approximately expressed as Equation (7).

(7) $$\psi \left(s\left(t\right)\right)\approx {\left(a\left(t\right)\psi^{\prime }\left(t\right)\right)}^{2}=a^{2}\left(t\right)\omega^{2}\left(t\right)$$

Meanwhile,

(8) $$\psi \left(s\prime \left(t\right)\right)\approx a^{2}\left(t\right)\omega^{4}\left(t\right)$$

Therefore, the envelope signal (instantaneous amplitude) |a(t)| obtained by demodulation and separation of signal s can be expressed as Equation (9):

(9) $$\left|a\left(t\right)\right|\approx \frac{\psi \left(s\left(t\right)\right)}{\sqrt{\psi \left(s^{\prime }\left(t\right)\right)}}$$

The signal energy in transmission is defined as the square of the amplitude of the signal. If the impact amplitude is small, the impact component may be drowned by other components. The Teager energy operator is the product of squared instantaneous amplitude and instantaneous frequency. Due to the high vibration frequency of transient impact, the Teager energy operator can effectively enhance transient impact components and significantly suppress noise.

In order to verify the superiority of the Teager energy operator spectrum (TEOS) compared with the envelope spectrum (ES) and the square envelope spectrum (SES), the following simulation signals are defined as Equation (10).

(10) $$y_{1}\;=\;\sum_{i=1}^{n}A_{}e^{-\xi {\left(t-q_{i}(t){/f}_{\mathrm{outer}}\right)}^{2}}\times \sin(2\pi f_{o}t)\;+\;n(t)$$

where *ξ* is the damping ratio, A is the amplitude, $q_{i}\left(t\right)\;=\;\left\lfloor t\times f_{i}\right\rfloor$ (i = 1, 2, …,n), $f_{o}$ is the natural frequency, $f_{\mathrm{outer}}$ = 25 Hz is the characteristic frequency and n(t) is the Gauss white noise. The parameters of the simulation signal are shown in Table 1.

**Figure 1:**
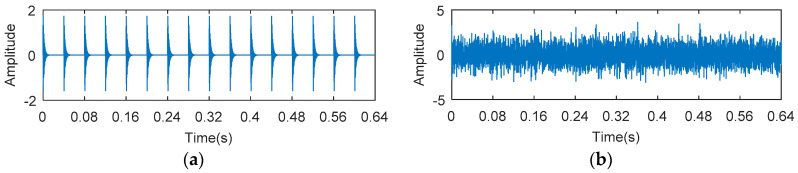
The simulated signals of (**a**) fault impulses; (**b**) fault impulses and noise (SNR = −12 dB).

**Figure 2:**
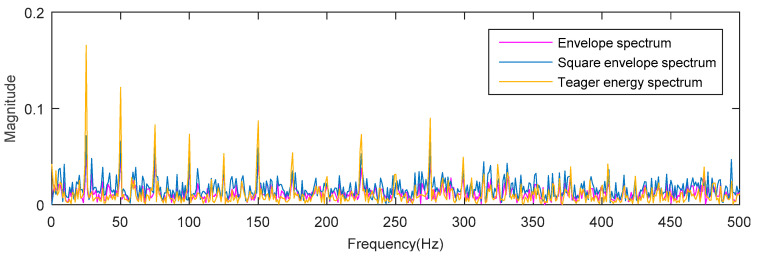
The analysis results of envelope spectrum, square envelope spectrum and Teager energy spectrum.

The waveforms of the synthetic signal with fault impulses and noise are shown in Figure 1, Figure 1a shows a periodic signal, which is used to simulate the fault impulses. Figure 1b shows a synthetic signal with fault impulses and noise (–12 dB). The envelope spectrum (ES), the square envelope spectrum (SES) and the Teager energy operator spectrum (TEOS) of the simulation signal are analyzed, respectively. The results are shown in Figure 2. It can be seen from Figure 2 that the amplitude of TEOS is higher than the amplitude of ES and the amplitude of SES at the fault characteristic frequency spectrum line. However, in the rest of the spectrum, the result is just the opposite. That is to say, the signal-to-noise ratio (SNR) of the Teager energy operator spectrum is much higher than that of the square envelope spectrum and the envelope spectrum.

### 2.2. Shannon Entropy

In 1948, Shannon put forward the concept of “Shannon entropy” and solved the problem of quantitative measurement of information. There is a direct relationship between the amount of information in a signal and its uncertainty, so from this point of view, we can think that the measure of the amount of information is equal to how much uncertainty there is. Shannon entropy reflects the degree of disordering (ordering) of a system, the more orderly a system is, the lower the entropy, and vice versa. In view of this, Shannon entropy can reflect the order of vibration signals. Given a random sequence $\left\{x_{1},\;x_{2},\;\ldots ,\;x_{n}\right\}$, the calculation method of Shannon entropy is shown in Equation (11):

(11) $$Sh=-c\sum_{i=1}^{n}p\left(x_{i}\right)\times \log\left(p\left(x_{i}\right)\right)$$

where Sh is the value of Shannon entropy, $p\left(x_{i}\right)$ is the probability mass associated with the value $x_{i}$ and c is an arbitrary positive constant that dictates the units.

**Figure 3:**
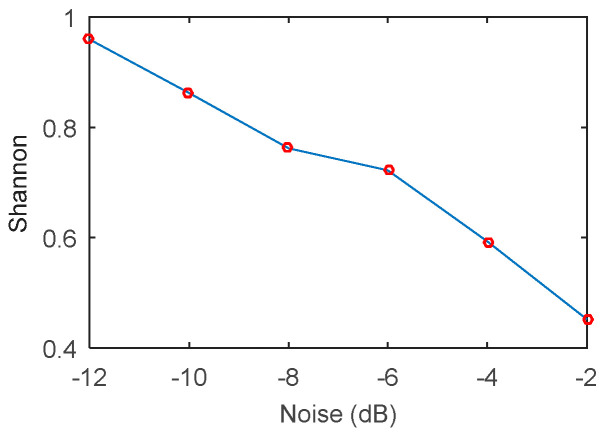
The curve of the Shannon entropy with different SNR.

The Shannon entropy index can reflect the regularity of the vibration signal, and its value has a certain relation with the SNR, so it can be used as an indicator to reflect the intensity of the impact of the fault. To verify this conclusion, the simulation signal model in Equation (10) is added with different degrees of noise to simulate the numerical changes of Shannon under different SNR. It can be seen from Figure 3 that the numerical regularity of Shannon entropy decreases with the increase of SNR.

### 2.3. Teager Energy Entropy

In view of the superiority of the Teager energy operator relative to the square envelope spectrum, we propose a Teager Energy Entropy (TEE) index. Inspired by infogram, we define the Teager energy entropy in the time domain (TEEt) and the Teager energy entropy in the frequency domain (TEEf) respectively as Equations (12) and (13).

(12) $$\Delta I_{\mathrm{TEEt}}=\frac{{\mathrm{TEO}}_{t}{\left(n;f,\Delta f\right)}^{2}}{\langle {\mathrm{TEO}}_{t}{\left(n;f,\Delta f\right)}^{2}\rangle }\mathrm{In}\left(\frac{{\mathrm{TEO}}_{t}{\left(n;f,\Delta f\right)}^{2}}{\langle {\mathrm{TEO}}_{t}{\left(n;f,\Delta f\right)}^{2}\rangle }\right)$$

(13) $$\Delta I_{\mathrm{TEEf}}=\frac{{\mathrm{TEO}}_{f}{\left(n;f,\Delta f\right)}^{2}}{\langle {\mathrm{TEO}}_{f}{\left(n;f,\Delta f\right)}^{2}\rangle }\mathrm{In}\left(\frac{{\mathrm{TEO}}_{f}{\left(n;f,\Delta f\right)}^{2}}{\langle {\mathrm{TEO}}_{f}{\left(n;f,\Delta f\right)}^{2}\rangle }\right)$$

In order to optimize the proportion of time domain and frequency domain components, an adaptive weighting method is proposed. Firstly, the regularization of TEEt and TEEf is carried out using Equations (14) and (15).

(14) $$T_{it}=\frac{\Delta I_{\mathrm{TEEt}i}-\overset{N}{\underset{i=1}{\min}}\Delta I_{\mathrm{TEEt}i}}{\overset{N}{\underset{i=1}{\max}}\Delta I_{\mathrm{TEEt}i}-\overset{N}{\underset{i=1}{\min}}\Delta I_{\mathrm{TEEt}i}}$$

(15) $$T_{if}=\frac{\Delta I_{\mathrm{TEEf}i}-\overset{N}{\underset{i=1}{\min}}\Delta I_{\mathrm{TEEf}i}}{\overset{N}{\underset{i=1}{\max}}\Delta I_{\mathrm{TEEf}i}-\overset{N}{\underset{i=1}{\min}}\Delta I_{\mathrm{TEEf}i}}$$

In these, *m* = 1, 2, …, *M* (*n* = 1, 2, …, *N*) represents the *M*(*N*)-th signal component. Then the mean value (μ) and standard deviation (σ) of the two regularization sequences are calculated separately using Equations (16)–(19).

(16) $$\mu_{t}=\frac{1}{M}\sum_{i=1}^{M}T_{it}$$

(17) $$\mu_{f}=\frac{1}{N}\sum_{i=1}^{N}T_{if}$$

(18) $$\sigma_{t}=\sqrt{\frac{1}{M}\sum_{i=1}^{M}{\left(T_{it}-\mu_{t}\right)}^{2}}$$

(19) $$\sigma_{f}=\sqrt{\frac{1}{N}\sum_{i=1}^{N}{\left(T_{if}-\mu_{f}\right)}^{2}}$$

Then the weight coefficients of the regularized sequence are calculated as Equations (20) and (21).

(20) $$C_{t}=\frac{\frac{1}{\sigma_{t}}}{\frac{1}{\sigma_{t}}+\frac{1}{\sigma_{f}}}$$

(21) $$C_{f}=\frac{\frac{1}{\sigma_{f}}}{\frac{1}{\sigma_{t}}+\frac{1}{\sigma_{f}}}$$

The Teager energy entropy, combined with the time domain and the frequency domain, is finally expressed as Equation (22):

(22) $$\mathrm{TEE}=C_{t}\Delta I_{\mathrm{TEEt}}+C_{f}\Delta I_{\mathrm{TEEf}}$$

### 2.4. Teager Energy Entropy Ratio of Wavelet Packet

**Figure 4:**
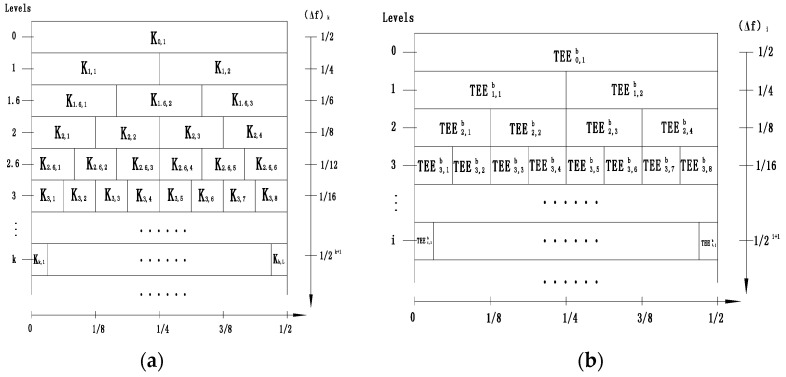
The paving of the (**a**) kurtogram and (**b**) TEEgram.

Lei et al. introduced wavelet packet transform (WPT) into the kurtogram algorithm to replace the traditional decomposition method (FIR, STFT) in kurtogram. Meanwhile, Stępień et al. confirmed that the entropy-based WPT is the most suitable approach that is not limited to one level of decomposition, and it allows us to find an optimal decomposition tree [17]. Inspired by this idea, the algorithm is further improved by the TEE index in this paper. The paving of the kurtogram and the TEEgram are shown in Figure 4.

**Figure 5:**
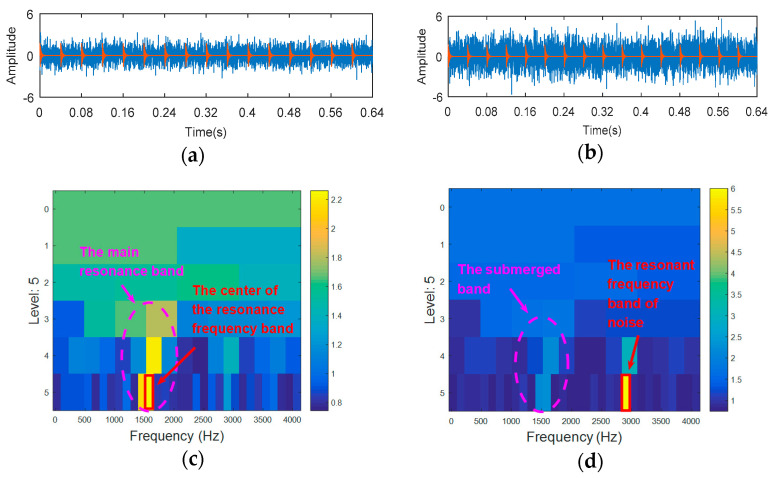
The processing results of TEEgram: (**a**) the synthetic signal of case 1 (SNR = −12 dB); (**b**) the synthetic signal of case 2 (SNR = −16 dB); (**c**) the TEEgram of (**a**); and (**d**) the TEEgram of (**b**).

In many cases, the components that reflect the characteristics of fault impact are likely to be drowned out by noise. This can easily cause misdiagnosis during the confirmation of the resonance frequency band. To illustrate these problems, different degrees of noise are added to the simulated signals in Equation (10). Figure 5a shows the simulated fault signal with SNR = −12 dB, Figure 5b shows the simulated fault signal with SNR = −16 dB, Figure 5c shows the TEEgram of (a), while Figure 5d shows the TEEgram of (b). When the signal-to-noise ratio (SNR) is −12 dB, the TEEgram can accurately locate the resonance frequency band (Table 1; the natural frequency is about 1500 Hz), but when the SNR is reduced to −16 dB, the resonance frequency band is inaccurate.

In view of the defect that kurtogram is too sensitive to strong background noise and singular points, Wang et al. proposed an improved method named SKRgram [18]. Subsequently, Miao et al. used the Gini coefficient as an index to further improve the SKRgram [19]. The essence of the SKRgram is to compare the change of spectral kurtosis before and after failure. Based on this, the potential bands of fault impact hidden in noise and other constant interference components can be found. Inspired by this idea, we further modify the TEE index as Equation (23):

(23) $$\mathrm{TEER}\;=\;{\mathrm{TEE}}_{failure}/{\mathrm{TEE}}_{health}$$

### 2.5. Process of the Proposed Method

**Figure 6:**
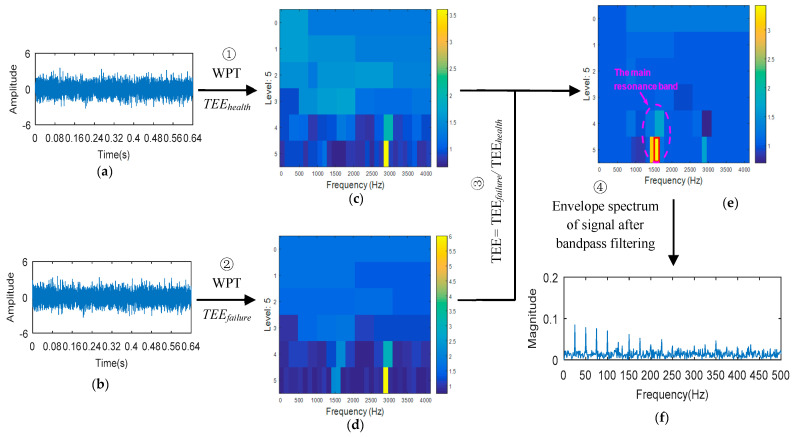
The process of the proposed method. (**a**) Healthy signal; (**b**) Fault signal; (**c**) TEEgram of the healthy signal; (**d**) TEEgram of the fault signal; (**e**) TEERgram; (**f**) Envelope spectrum.

1. The bearing vibration signals are collected under healthy state and fault state conditions (Figure 6a,b). According to the bearing parameters, the characteristic frequency of bearing fault is calculated based on the theoretical equations (Equation (24)).
  (24) $$\begin{array}{l} f_{i}=\frac{Z}{2}(1+\frac{d}{D}\cos\beta )\frac{N}{60},\;f_{o}=\frac{Z}{2}(1-\frac{d}{D}\cos\beta )\frac{N}{60}\\ f_{\mathrm{ball}}=\frac{D}{2d}(1-(\frac{d}{D}{)}^{2}\cos^{2}\beta )\frac{N}{60},\;f_{\mathrm{cage}}=\frac{1}{2}(1-\frac{d}{D}\cos\beta )\frac{N}{60} \end{array}$$
  where $f_{i}$, $f_{o}$, $f_{\mathrm{ball}}$ and $f_{\mathrm{cage}}$ respectively represent inner ring fault, outer ring fault, rolling element fault and cage fault. d is the ball diameter, D is the pitch diameter, β is the contact angle, N is the speed of rotation, Z is the number of the balls.
2. Wavelet packet transform (WPT) is applied, in turn, to vibration signals in healthy state and fault state. The time domain Teager energy entropy ($\Delta I_{\mathrm{TEEt}}$) and the frequency domain Teager energy entropy ($\Delta I_{\mathrm{TEEf}}$) are calculated for each sub-band of the signal under the healthy condition. $\Delta I_{\mathrm{TEEt}}$ and $\Delta I_{\mathrm{TEEf}}$ are weighted to obtain Teager energy entropy (${\mathrm{TEE}}_{health}\;=\;C_{t}\Delta I_{\mathrm{TEEt}}\;+\;C_{f}\Delta I_{\mathrm{TEEt}}$) in the healthy state. Similarly, the energy entropy of Teager (${\mathrm{TEE}}_{failure}$) under the fault condition is obtained. Teager energy entropy diagram (TEEgram) of wavelet sub-band of health status and fault state signals is constructed respectively (Figure 6c,d).
3. According to the Teager energy entropy of the signal in the healthy state (${\mathrm{TEE}}_{health}$) and the Teager energy entropy of the signal in the fault state (${\mathrm{TEE}}_{failure}$), the Teager energy entropy rate ($\mathrm{TEER}\;=\;{\mathrm{TEE}}_{failure}/{\mathrm{TEE}}_{health}$) is calculated. Teager energy entropy ratio diagram (TEERgram) of wavelet sub-band is constructed as shown in Figure 6e. It can be seen from Figure 6e that the location of the relative change of TEE value is the fault resonance frequency band. The wavelet subband with the largest TEER value is selected as the optimal band. The center frequency and bandwidth are extracted to set the parameters of the bandpass filter, and the filter is used to filter the selected wavelet sub-bands. Then the envelope spectrum of the filtered signal is analyzed (Figure 6f). The fault type is analyzed by comparing the characteristic frequency of the spectrum line with the theoretical characteristic frequency calculated in step 1.

## 3. Simulation Analysis

The effectiveness of the proposed algorithm is verified by the mathematical model of the inner ring fault simulation signal in Equation (25) [20].

(25) $$\left\{ \begin{array}{l} x(t)=s(t)+n(t)=\sum_{i}^{}A_{i}h(t-iT-\tau_{i})+n(t)\\ A_{i}=A_{0}\cos(2\pi f_{r}t+\phi_{A})\\ h(t)=e^{-Bt}\cos(2\pi f_{n}t+\phi_{\omega }) \end{array}\right.$$

where $\tau_{i}$ is the ith shock; T is the period of shock; $f_{r}$ is the rotating frequency; $A_{i}$ is the amplitude modulation with $1/f_{r}$ as a cycle; h(t) is an exponential decay pulse; B is the attenuation coefficient of vibration caused by damping; and $\phi_{A}$, $\phi_{\omega }$ represent the phase. The other parameters of the simulation signal are shown in Table 2.

**Figure 7:**
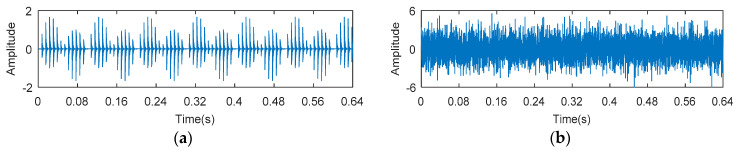

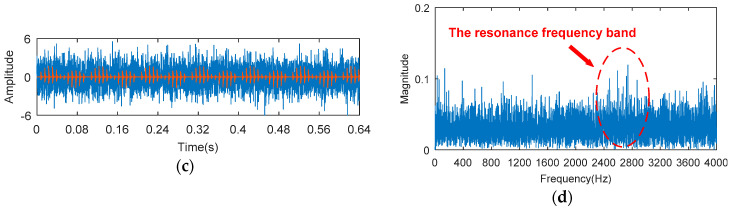
The simulated signals of (**a**) the inner fault impulses; (**b**) the noise signal; (**c**) the synthetic signal (SNR = −16 dB) and (**d**) the spectrum of (**c**).

Figure 7a shows the waveform of the inner fault impulses; Figure 7b shows the noise; Figure 7c shows the synthetic signal with fault impulses and noise (−16 dB). Figure 7d shows the spectrum of Figure 7c. It can be seen from Figure 7d that the fault feature information cannot be found, and there is a suspected resonance frequency band near 2500 Hz.

**Figure 8:**
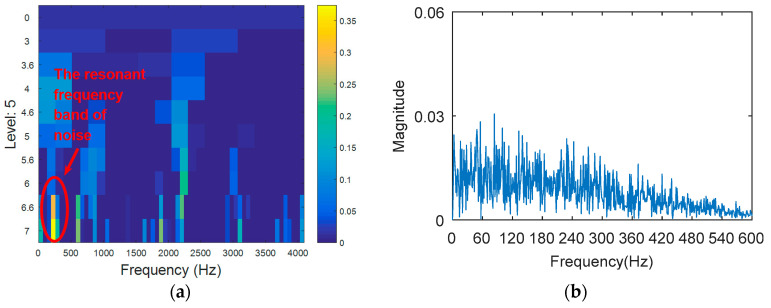
The processing results of kurtogram: (**a**) the kurtogram; and (**b**) the envelope spectrum of the selected band.

**Figure 9:**
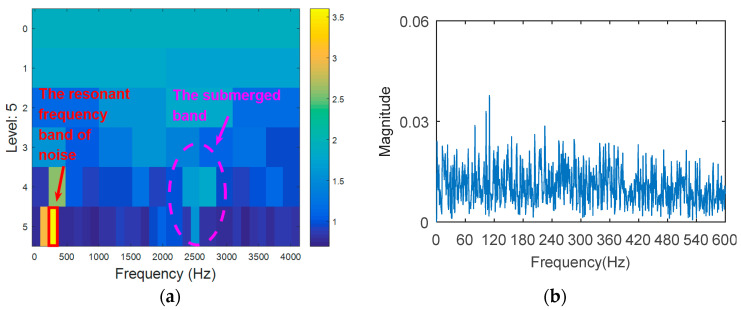
The processing results of KWPT: (**a**) the kurtogram; and (**b**) the envelope spectrum of the selected band.

**Figure 10:**
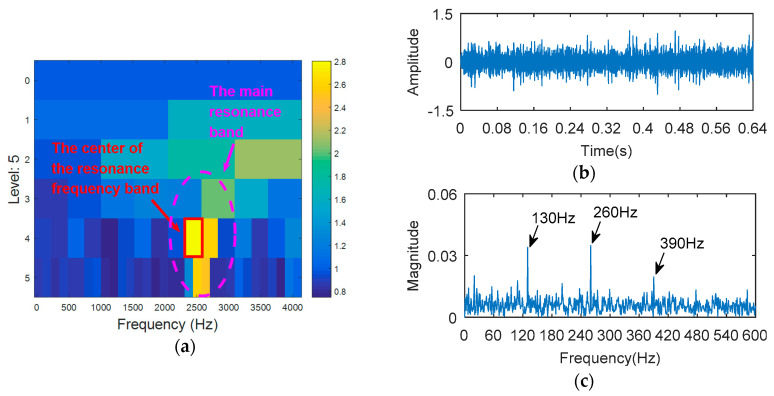
The processing results of proposed method: (**a**) the TEERgram; (**b**) the filtered signal and (**c**) the envelope spectrum of (**b**).

The simulation signal is respectively analyzed by kurtogram, kurtosis of the wavelet packet transform (KWPT) and the proposed method. The results of kurtogram analysis are shown in Figure 8. The results of KWPT analysis are shown in Figure 9. The two methods are inaccurate in locating the resonant frequency band, and are significantly affected by noise. In the envelope spectrum of their sub-bands, the characteristic frequencies of faults can be found. Figure 10 is the method proposed in this paper. By calculating the Teager energy operator ratio (TEER) of the signal before and after the fault, the resonant frequency band that changes before and after the fault is obtained, that is, the resonance frequency band caused by the fault.

## 4. Experiment Analysis

### 4.1. Experiment 1

**Figure 11:**
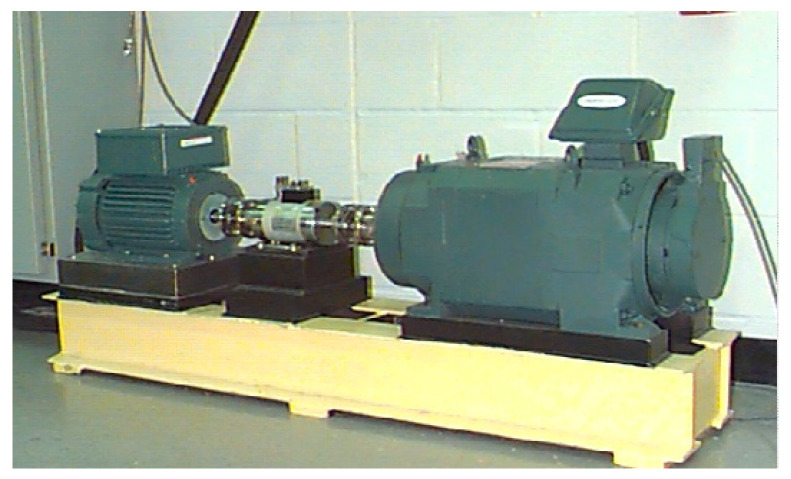
The bearing test stand at Case University.

Data from the bearing data center, Case Western Reserve University, are widely used in the field of fault diagnosis as common open data, and have a high degree of recognition (Figure 11) [21]. The experimental data with the weakest degree (0.007 inch) of failure are used to verify the effectiveness of the proposed method in experimental applications. The sampling frequency is 12 kHz, and the rotation speed is 1772 r/min. The bearing parameter dimensions (JEMSKF6023-2RS) are shown in Table 3, and the characteristic frequency of the bearing fault calculated by the theoretical formula is shown in Table 4.

**Figure 12:**
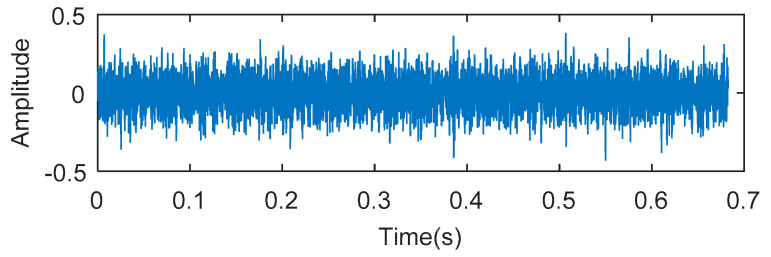
The experimental signal of Experiment 1.

**Figure 13:**
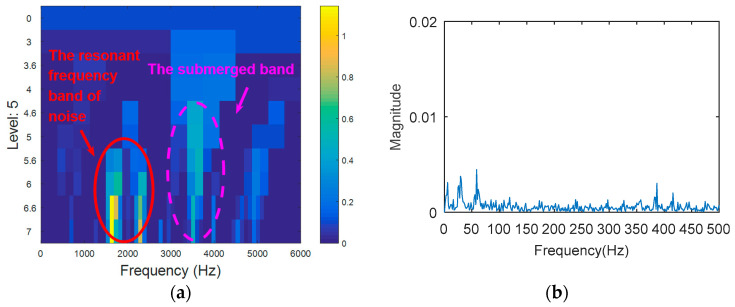
The processing results of kurtogram: (**a**) the kurtogram; and (**b**) the envelope spectrum of the selected band.

**Figure 14:**
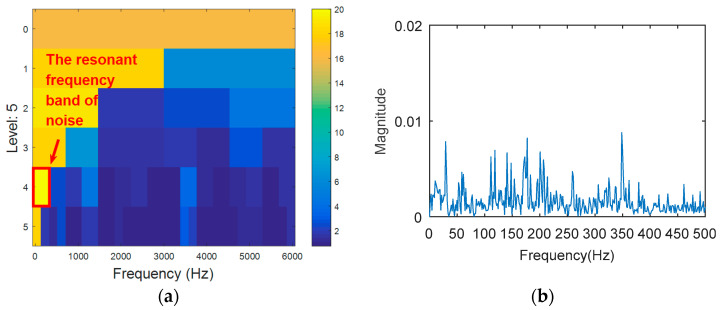
The processing results of KWPT: (**a**) the kurtogram; and (**b**) the envelope spectrum of the selected band.

**Figure 15:**
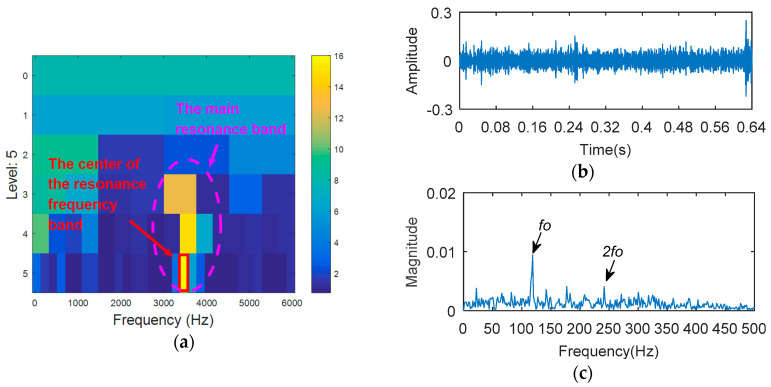
The processing results of proposed method: (**a**) the TEERgram; (**b**) the filtered signal, and (**c**) the envelope spectrum of (**b**).

8192 points of vibration signal of rolling element fault (Figure 12) are analyzed. The vibration signal of rolling element fault is respectively analyzed by kurtogram, KWPT and the proposed method. The results of kurtogram analysis are shown in Figure 13. The results of KWPT analysis are shown in Figure 14. The two methods are inaccurate for locating the resonant frequency band, and are significantly affected by noise. In the envelope spectrum of their sub-bands, the characteristic frequencies of faults can be found. Figure 15 shows the method proposed in this paper. The center of the resonance band determined by the TEEgram is about 3500 Hz. The partial band is filtered, and then the envelope spectrum of the filtered signal is analyzed. It can be seen from the envelope spectrum (Figure 15c) that the fault characteristic frequency and its first harmonic are effectively extracted.

### 4.2. Experiment 2

**Figure 16:**
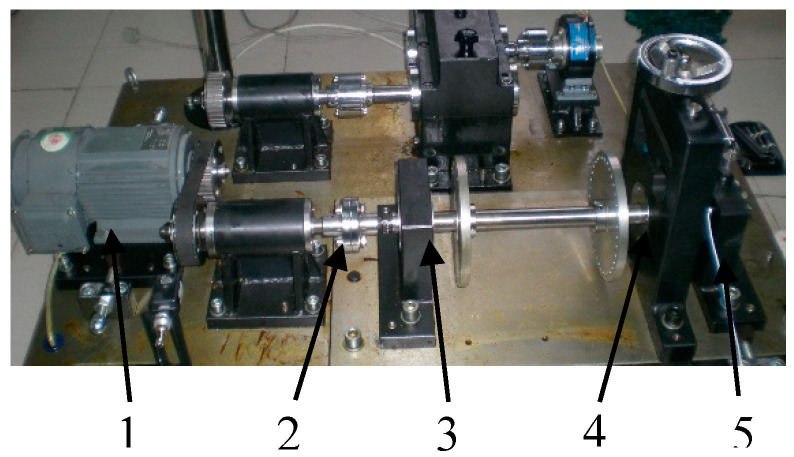
The experiment platform of QPZZ-II. 1. The drive motor; 2. The coupling; 3. The bearing housing with the normal bearing seat; 4. The loading device; and 5. The bearing housing with the fail bearing seat.

**Figure 17:**
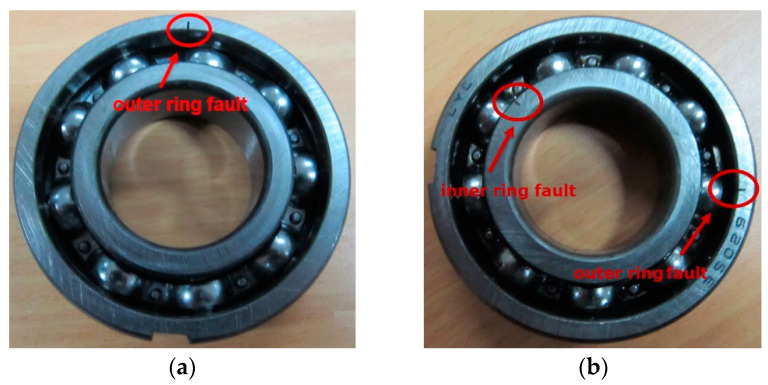
Bearing with (**a**) outer ring fault; and (**b**) inner and outer compound fault.

Experiment 2 is designed on the experiment platform to further verify the robustness of the proposed method. Experiment 2 contains two parts, which are the outer ring fault experiment and the inner and outer ring compound fault experiment. The bearing failure experiments are carried out on the QPZZ-II rotating machinery failure experiment platform. The structure of the experiment platform is shown in Figure 16. The type of bearing used in the experiment is LYC6205E (Figure 17). The parameters of the rolling bearing are shown in Table 5. The spark erosion technique is used to process faults in inner rings and outer rings of bearings. The degrees of failure are 1.5 mm deep and 0.2 mm wide. The acceleration sensor arranged on the bearing seat is used to collect vibration signals generated during the rotation of the bearing. The sampling frequency is 12,800 Hz, and the motor speed is 1466 r/min.

The characteristic frequency of inner ring and outer ring fault can be calculated from the following equations:

$$f_{o}=\frac{Z}{2}(1-\frac{d}{D}\cos\alpha )\frac{N}{60}=87.7\;\mathrm{Hz},\;f_{i}=\frac{Z}{2}(1+\frac{d}{D}\cos\beta )\frac{N}{60}=132.2\mathrm{Hz}$$

#### 4.2.1. Case 1: Analysis of Outer Ring Fault

**Figure 18:**
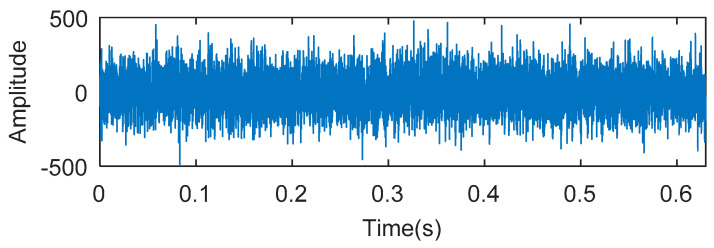
The experimental signal of outer ring fault in experiment 2.

8192 points of vibration signal of outer ring fault (Figure 17a) are analyzed, and the time domain waveform of the outer ring fault is shown in Figure 18.

**Figure 19:**
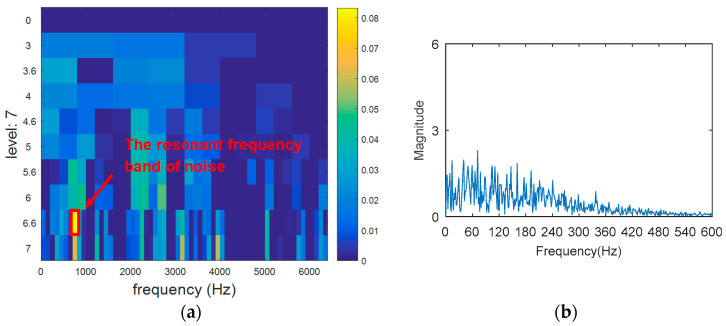
The processing results of kurtogram: (**a**) the kurtogram; and (**b**) the envelope spectrum of the selected band.

**Figure 20:**
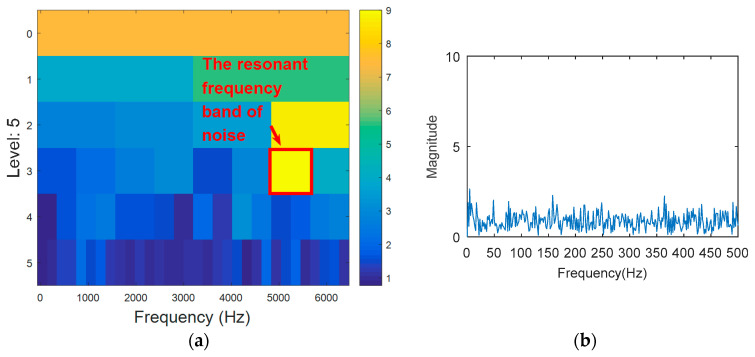
The processing results of KWPT: (**a**) the kurtogram; and (**b**) the envelope spectrum of the selected band.

**Figure 21:**
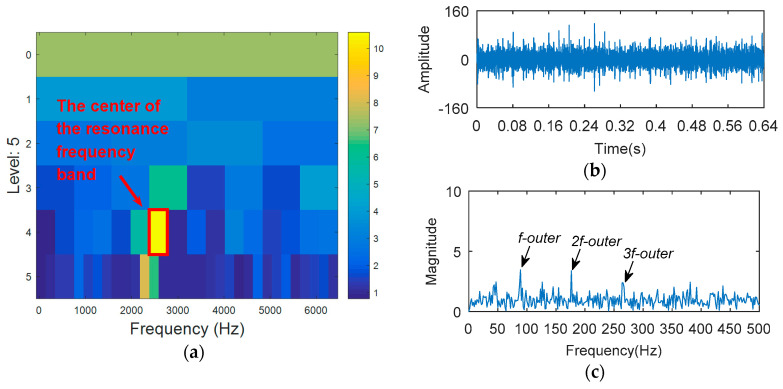
The processing results of the proposed method: (**a**) the TEERgram; and (**b**) the filtered signal; and (**c**) the envelope spectrum of (**b**).

The outer ring fault signal is analyzed via the kurtogram (Figure 19). As shown in Figure 19a, the central frequency of the resonant band determined by the kurtogram method is 860 Hz. The band pass filtering is applied to the resonant frequency band and envelope spectrum analysis of filtered signals is carried out. As shown in Figure 14b, fault feature information is not found in the envelope spectrum. This means that the resonant frequency identified by kurtogram does not contain fault feature information; that is, kurtogram is not accurate at locating the resonant frequency band. Similarly, the resonant frequency band in Figure 20a is filtered and envelope spectrum analysis is performed, and the fault characteristic spectrum is not found in Figure 20b. This indicates that the KWPT method is also invalid. Finally, the vibration signal is analyzed by the proposed method. The center of the resonant frequency band is about 2600 Hz from the TEERgram shown in Figure 21a. The envelope spectrum analysis of the band is shown in Figure 21c. The characteristic frequency and its harmonics of the bearing outer ring fault can be found.

#### 4.2.2. Case 2: Analysis of Compound Fault in Inner and Outer Ring

**Figure 22:**
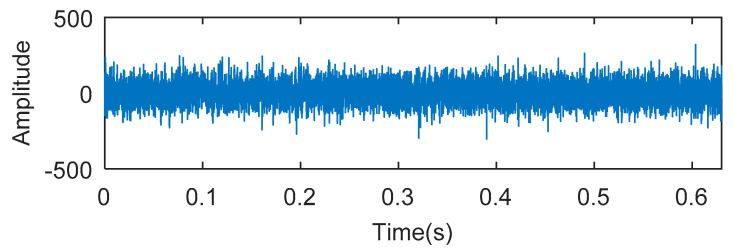
The experimental signal of inner and outer ring fault in Experiment 2.

**Figure 23:**
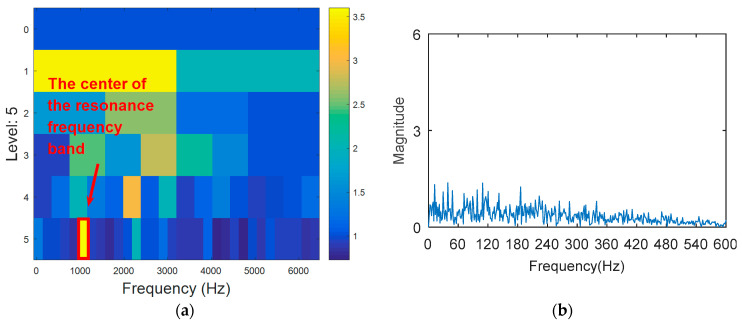
The processing results of KWPT: (**a**) the kurtogram; and (**b**) the envelope spectrum of the selected band.

**Figure 24:**
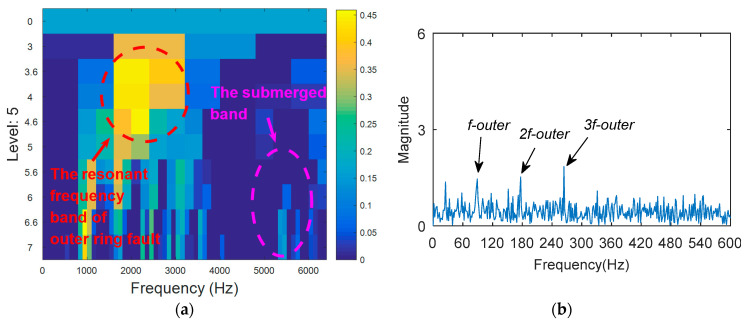
The processing results of kurtogram: (**a**) the kurtogram; and (**b**) the envelope spectrum of the selected band.

**Figure 25:**
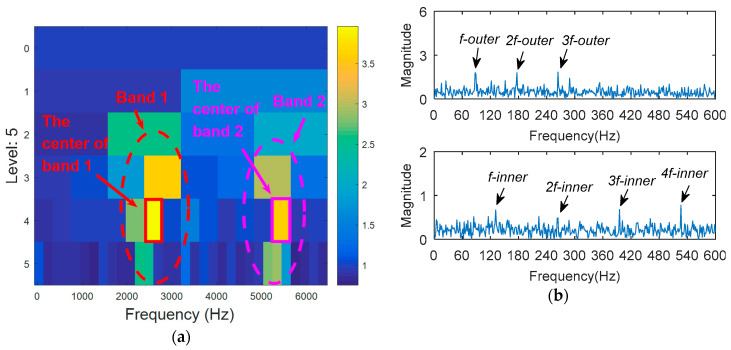
The processing results of proposed method: (**a**) the TEERgram; and (**b**) the envelope spectrum of the selected band.

8192 points of vibration signal of inner and outer ring fault are analyzed (Figure 17b), and the time domain waveform is shown in Figure 22. Figure 23 shows the processing result of KWPT. It can be seen that due to noise interference, the sub-band determined by KWPT is a sub-band containing more noise, rather than a sub-band containing more fault feature information. The sub-band containing the characteristic information of the outer ring fault is not obvious, and the sub-band containing the fault information of the inner ring is completely submerged. The kurtogram analysis of compound fault vibration signal is shown in Figure 24. It can be seen from Figure 24a that the resonant frequency band of the outer ring fault is found, but the resonant frequency band of the relatively weak inner ring fault is submerged. The resonance frequency band is filtered, and the characteristic frequency and frequency doubling of the outer ring fault can be found in the envelope spectrum shown in Figure 24b, but there is no characteristic information of the inner ring fault. Then, the composite fault signal is analyzed by the method proposed in this paper. With the benefits of the relative change rate of the resonance band before and after failure, the main frequency band and the secondary frequency band can be found in the TEERgram in Figure 25a. The two frequency bands are filtered respectively, and the envelope spectrum analysis of filtered signals are shown in Figure 25b. The fault characteristic frequency and its harmonics of inner ring and outer ring fault are separated and extracted. The advantage of TEERgram is that the ratio of Teager energy entropy before and after fault is calculated as an index. This ratio reflects the relative change value caused by faults, not the size of the absolute quantity; that is, the change caused by the failure. This will avoid the influence of the absolute amount caused by random impulses and noise to the analysis process, and to a certain extent, it avoids the influence of stronger faults on the weaker faults. Therefore, we think that this method is effective for single faults submerged in random impulses and noise, and also has certain effect for some compound faults.

## 5. Conclusions

In this paper, the TEERgram method is proposed. On one hand, WPT is used to replace the STFT and FIR filters in traditional kurtogram. WPT can divide the frequency band meticulously and suppress noise at the same time. On the other hand, the TEER is used as the computing index of the wavelet sub-band to find the optimal resonance frequency band. The time domain entropy ($\Delta I_{\mathrm{TEEt}}$) and frequency domain entropy ($\Delta I_{\mathrm{TEEf}}$) of wavelet sub-bands are processed statistically so that the TEE index can reflect the impact characteristics and periodic characteristics adaptively. By calculating the change of the TEER value before and after the fault, it can highlight the effect of the fault. Finally, simulations and experiments are carried out to demonstrate the effectiveness of the proposed method and its superiority over kurtogram.

## Figures and Tables

**Table 1:** Parameters of the simulated signal.

Amplitude	Rotating Frequency $f_{r}$ (Hz)	Natural Frequency (Hz)	Sampling Frequency (Hz)	Fault Frequency (Hz)
2	20	1500	8192	25

**Table 2:** Parameters of the inner fault simulation.

Amplitude	Rotating Frequency $f_{r}$ (Hz)	Natural Frequency (Hz)	Sampling Frequency (Hz)	Fault Frequency (Hz)
2	20	2500	8192	130

**Table 3:** Structure parameters of the inner fault simulation.

Bearing Type	Pitch Diameter (mm)	Ball Diameter (mm)	Number of Balls	Contact Angle (°)
SKF6023-2RS	28.5	6.7	8	0

**Table 4:** Fault feature frequency of the rolling bearing.

Types of Failures	Outer Ring	Inner Ring	Rolling Element
Defect frequencies/Hz	90	145	118

**Table 5:** Structure parameters of the inner fault simulation.

Bearing Type	Pitch Diameter (mm)	Ball Diameter (mm)	Number of Balls	Contact Angle (°)
LYC6205E	39	7.9	9	0
